# Role of Telemedicine and Digital Technology in Public Health in India: A Narrative Review

**DOI:** 10.7759/cureus.35986

**Published:** 2023-03-10

**Authors:** Revathi G Maroju, Sonali G Choudhari, Mohammed Kamran Shaikh, Sonali K Borkar, Harshal Mendhe

**Affiliations:** 1 Department of Community Medicine, Datta Meghe Medical College, Datta Meghe Institute of Medical Sciences, Wardha, IND; 2 School of Epidemiology & Public Health; Department of Community Medicine, Jawaharlal Nehru Medical College, Datta Meghe Institute of Medical Sciences, Wardha, IND

**Keywords:** telehealth, telediagnosis, remote consultation, telecommunication, digital health, covid-19 pandemic

## Abstract

There are still many areas of India without proper medical facilities. In such a setting, technology can play a facilitating role, particularly in reaching out to remote locations and offering a greater standard of care at a lower cost. The method of treating and diagnosing patients remotely through communication networks is known as telemedicine. When more patients get access to telemedicine, payers take more notice of how much less expensive it is than traditional medicine, and doctors are aware of its benefits. Telemedicine is a more beneficial technology that can expand access to preventive treatment and may lead to long-term health. Telemedicine has the potential to greatly affect public health. This paper reviews the current state of the art of telemedicine in India. Nearly 50 years ago, telemedicine was shrugged off as a complicated, expensive, and inefficient technology. Because of how quickly the information technology and telecommunications disciplines are advancing, telemedicine is today a viable, dependable, and useful technique. Practitioners and medical experts from a variety of fields have experienced success with telemedicine. The COVID-19 pandemic highlighted the need for strong primary healthcare networks for a more effective public health response during health emergencies and exposed the fragmentation of healthcare delivery systems. Although primary care is the first point of contact between the general public and the healthcare system, it has not recently grown much focus or funding. Even in the post-COVID-19 environment, telemedicine offers the potential to get through enduring barriers to primary care in India, such as a shortage of qualified medical professionals, issues with access, and the cost of in-person care. Telemedicine has the power to speed up the delivery of universal health coverage while strengthening primary care. There is a widening gap between people and those who offer basic health services as the population in India has grown, and the average lifespan has increased. Telemedicine helps with palliative care, early identification, a better cure, prevention, and rehabilitation in the treatment of cancer. Due to a shortage of primary care delivery networks and referral units, secondary and tertiary care facilities' health systems are overworked. To successfully use telemedicine, proper planning and operating processes are required. Thus, the development and implementation of telemedicine will improve patient care and India's primary healthcare system in the future. Finally, telemedicine's cost-effectiveness will likely be its most significant outcome.

## Introduction and background

Telemedicine is the "natural evolution of healthcare in the digital world," according to the American Telemedicine Association [1]. According to the WHO, "the delivery of healthcare, where distance is a critical factor, by all medical professionals using information and communications technology for the exchange of valid information for the diagnosis, treatment, and prevention of disease and injuries, research and evaluation, and for the ongoing training of health care providers, all in the best interest of advancing the health of individuals and communities" is broadly referred to as telemedicine [2]. Telemedicine is the technique of treating and diagnosing patients online from anywhere in the globe using communication networks. More people use telemedicine as payers notice the lower cost of treatment, physicians understand the advantages, and patients have more access to it [3]. People may increase healthcare delivery and make it available to more individuals by using technology and the best network services. A more advantageous technology is telemedicine, which may improve long-term health and increase access to preventive care. This particularly benefits individuals who struggle to find adequate treatment because of their financial circumstances or geographical condition [4]. Therefore, it is possible to think about expanding teleconsultation methods to ease the load on hospitals and promote a safer working environment for healthcare professionals [5].

The National Medical College Network, the Digital Medical Library Network, the National Cancer Network, and the National Rural Telemedicine Network are a few of the initiatives that the Ministry of Health of the government of India has started to work on to advance the cause. By sending and receiving precise data for the diagnosis, treatment, and prevention of disease and injuries, research and evaluation, as well as for the ongoing education of healthcare professionals, telemedicine is an essential tool that can address one such imbalance and improve the delivery of healthcare [6]. The smartphone-driven digital revolution has advanced all facets of life over the past 10 years, including healthcare [7]. The rapid growth in the usage of smartphones, coupled with its equivalents in tablet technology and wearables like smartwatches, marked the beginning of this revolution. These have been developed to include wearable sleep technologies, patient electrocardiogram recording applications, and the ability to identify falls in elderly patients [8-10]. Telemedicine was made possible by such programs and high-speed internet connections, which allowed for efficient text, voice, and video connections between patients and their doctors in a matter of seconds [11].

Methodology

**Figure 1:**
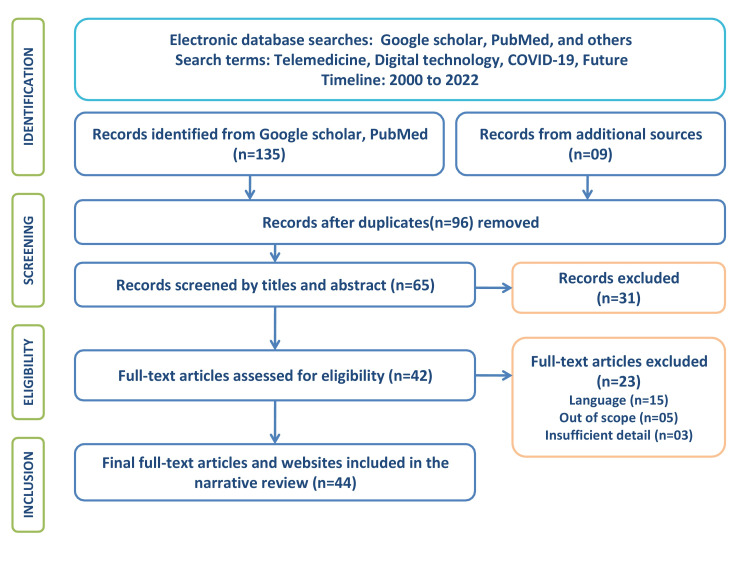
Flow diagram of literature search and inclusion criteria

A literature review was conducted using Google Scholar, Pub Med, and other databases to find articles published between 2000 and 2022. The words "telemedicine," "digital technology," "COVID-19," and "future" were used. The inclusion criteria were publications between 2000 and 2022, published works that are in English, and articles related to telemedicine in all contexts. We removed duplicates, abstracts, works that were written in languages other than English, unpublished works, and resources that didn't have much to do with telemedicine. Figure 1 depicts the Preferred Reporting Items for Systemic Reviews and Meta-Analyses (PRISMA) method followed for the literature analysis.

## Review

Historical outlook of telemedicine

Telemedicine's value is its ability to transfer medical data over long distances. In the first decade of the twentieth century, telemedicine was first recorded in a published report as electrocardiogram transmission over telephone lines [1]. Maybe the earliest known form of telemedicine was the transmission of information about health-related events like outbreaks and epidemics using ancient hieroglyphic writing and scrolls [12]. Telemedicine was dismissed nearly 50 years ago because it was a cumbersome, unreliable, and expensive technology. Telemedicine is now a viable, dependable, and practical technique because of the rapidly developing telecommunications and information technology fields. Many different medical specialties and practitioners have reported success with telemedicine [13]. 

The first telemedicine service in India was established by Apollo Hospital in the Andhra Pradesh village of Aragonda in the Chittoor District. It was linked to Apollo Hospital in Chennai through telemedicine. Mammography services at Sri Ganga Ram Hospital in Delhi, oncology at Regional Cancer Center in Trivandrum, and surgical services at Sanjay Gandhi Postgraduate Institute of Medical Sciences in Lucknow are a few noteworthy examples of the successful implementation of telemedicine services in India [6].

Need for telemedicine

One of the most essential issues in giving the poor access to high-quality healthcare is the technology used in telemedicine, which enables clinicians and patients to be nearly anywhere. Due to the development of telemedicine, access to healthcare in rural areas is no longer hampered by distance [1]. Poverty, a lack of development, and civil upheaval have an especially negative influence on India's North Eastern states [14]. The region's subpar infrastructure and low connectivity to the rest of the country are the main obstacles to the growth and development of these states. Through the use of telemedicine and digital pathology, many patients in these areas can obtain local care while consulting with specialists in larger cities [15]. Telemedicine technology, which might be incorporated into the current healthcare delivery system, might be used to lessen the disparity in access to healthcare between rural and urban locations. In rural and suburban India, it is much easier to build a communications infrastructure than it is to recruit hundreds of doctors [14, 16]. In India, 90% of secondary and tertiary healthcare services are located outside of rural areas, where 68% of the population lives. There are very few adequate primary healthcare facilities for the rural population. Despite government and private sector attempts, the lack of access to high-quality healthcare in rural and distant locations continues to be a problem [15]. 

Newest trends of telemedicine in India

The use of digital pathology for routine diagnoses is rapidly expanding. This improvement was made possible by faster full-slide picture scanning, but it is difficult to implement on a big scale from a technological, practical, and financial aspect [15]. The field of medicine can still be actively affected by telemedicine from time to time. Medical benefits and value benefits are the two main areas where this innovation has advantages. The increase in technical and technological aspects of healthcare may be a logical extension of telemedicine [16]. The medical benefits of effective telemedicine programs are related to how experts utilize the technology. The medical impact of telemedicine is summarized by a modified version of a famous analogy from the instructional analysis that was formerly used for telemedicine [17]. Despite evidence indicating a significant decrease in the use of primary care for non-emergency and non-COVID-19 conditions, such as chronic medical disorders, during the pandemic, teleconsultations have become more common. This suggests that handling emergencies has been telemedicine's primary goal. In nations with pre-existing regulatory frameworks and supportive national digital health frameworks, telehealth interventions improved primary care healthcare while also enhancing the public health response to COVID-19 [18]. 

These telemedicine initiatives must be used more frequently, even after the pandemic crisis, to improve the quality, equity, and training of Indian healthcare services. Success depends on establishing priorities for short-, medium-, and long-term objectives. In the long term, it is essential to improve internet infrastructure [19]. Together with government initiatives to supply optic fiber to rural areas, connecting the smallest administrative and healthcare organizations, such as public health centers and health and wellness centers, with larger hospitals and medical college hospitals is crucial. The intermediate period requires objective assessment to be important. It is time to evaluate present telemedicine initiatives, both public and private. The evaluation's results need to influence changes to the law and legal system. The critical need shortly is for medical practitioner training. Continuing medical education modules or "crash courses" in telemedicine can help practitioners stay current with technological, ethical, and legal issues by bringing awareness to their patients. Gains in telemedicine use in India are anticipated to continue, creating the ideal conditions for a more effective healthcare delivery system [20].

Many practitioners were forced to enter the telemedicine field for the first time as a result of the COVID-19 outbreak. Limiting patient interaction and contacts could reduce transmission because several otolaryngology procedures could produce aerosolized virus particles. Recent changes to Medicare fee-for-service billing, which enabled equal compensation for virtual consultations, encouraged telemedicine as an economically viable and moral response to the epidemic [21]. 

With the help of telemedicine and direct care, the Chunampet Rural Diabetes Prevention Initiative aims to provide comprehensive diabetes screening, prevention, and treatment in rural India [22]. In India, telemedicine programs are actively promoted by the Indian Space Research Organization, the Department of Information Technology, the Asia Heart Foundation, state governments, Apollo Hospitals, and a few other non-governmental sectors. Apollo Telemedicine Enterprises, Narayana Hrudayalaya, Escorts Heart Institute, Asia Heart Foundation, and Aravind Eye Care are a few of the top telemedicine rivals in India at the moment [6]. A tele-oncology network exists in Kerala for more than 10,000 cancer patients annually, who regularly use this communication to keep up with their onco-physician. During Maha Kumbhamelas and other festivals, the Uttar Pradesh government also uses telemedicine [23]. Telemedicine has evolved into a form of "forward triage" in which patients are evaluated even before attending an emergency room, in response to the COVID-19 outbreak. One technique for screening patients despite their self-quarantine has arisen, and it is called direct-to-consumer telemedicine or on-demand telemedicine [24]. By ensuring patient-centered care, this triage technique protects both patients and medical personnel. The quick response to COVID-19 infection may include respiratory symptoms, which have been evaluated using telemedicine in individuals who may be infected [25].

According to a study, patients now view telemedicine as advantageous and appropriate for providing healthcare services during the COVID-19 pandemic. The patients are more willing to try telemedicine now, which will result in higher acceptability of telemedicine in the post-COVID era. The patients identified economic and travel savings as secondary reasons for choosing telemedicine services. Last but not least, the majority of people now require telemedicine in some way. They are making an effort to adopt technology without being scared of it [26].

Applications

Telemedicine has several potential applications, including:

Tele-Education

Distance learning is possible with the use of telecommunication technologies. It is also incredibly interactive and versatile. An adaptable and engaging long-distance learning program that offers more convenient training and updates on the most recent developments for more precise and efficient treatment methods [1].

Remote Consultation

Telehealthcare can be used for long-distance medical service delivery, promotion, and prevention. It could take the form of a consultation or follow-up. Telemedicine has also been helpful to meet the problem of providing healthcare at large Indian gatherings. For instance, during Maha Kumbhamelas, the government of Uttar Pradesh utilizes mobile telemedicine vans fully equipped with videoconferencing systems for visual communication. This enables doctors in remote locations to connect to any hospital that offers telemedicine services, including super specialty hospitals, for expert advice [6].

Disaster Management

Telemedicine has the potential to significantly assist with both natural catastrophes like earthquakes, tsunamis, and tornadoes and man-made disasters like war and riots. A transportable and portable telemedicine system with satellite connectivity and specialized telemedicine software is appropriate for disaster assistance as the bulk of terrestrial communication lines either do not function enough or break during disasters [23].

Tele-Home Healthcare

Patients who are old or underserved and restricted to their homes due to chronic conditions may receive in-home care using telemedicine technology. It enables home healthcare professionals to keep an eye on patients from a central location rather than traveling to distant regions to check on recovering or chronically unwell patients. A more cost- and time-efficient substitute is remote patient monitoring [1].

Advantages and drawbacks

There are several advantages of telemedicine, such as removing geographical boundaries and delivering healthcare services to remote and rural areas; it helps the population who live in isolated areas (health for all). It also removes barriers brought on by distance and expands the availability of excellent medical care. Moving a patient would be unwanted or impractical in situations involving emergency and critical care; therefore, it is especially useful in these instances. Receiving professional care and support is made simpler for patients and distant medical professionals, thanks to telemedicine. Additionally, it reduces the cost and/or difficulty of patient transfers as well as the needless travel time for medical staff. It also lessens the isolation of rural practitioners by improving their understanding through tele-education or tele-continuing medical education [27].

Age, race, place of residence, payer, and telehealth use vary widely during the COVID-19 Public Health Emergency's initial phase of telehealth expansion. More research is required to further understand the underlying reasons for these differences and to influence policy decisions both during and after the COVID-19 emergency [28]. Telemedicine benefits include flexible work schedules, decreased travel time, high patient satisfaction, increased access to high-quality specialist treatment, and cost savings for patients and healthcare systems [21]. The "tools" of telemedicine have enhanced telemedicine consultations by making them more data-based and scientific. Examples include digital stethoscopes and otoscopes, oxygen saturation probes (to measure the patient's oxygen level), and blood pressure monitors [29]. The major benefit of telemedicine is that it allows the doctor to visit areas that have never been visited by a doctor before, and telemedicine genuinely has the power to transform lives in a huge country like India where sizable portions of the population lack access to doctors [30, 31].

Cancer and other non-communicable illnesses are becoming significant public health issues in India. Given the rising frequency of cancer in the nation, raising national awareness of cancer and its care is given the utmost attention. Through the use of contemporary communication and information technologies, telemedicine offers expert-based healthcare to understaffed remote locations and cutting-edge emergency treatment. If people are informed about cancer and its underlying causes, the incidence of cancer can be decreased. The advent of telemedicine in oncology recently has proven outcomes. In the management of cancer, telemedicine aids in palliative care, early detection, a better cure, prevention, and rehabilitation [32].

Telemedicine has its difficulties, too. The majority of people would agree with this, yet there are still individuals who only ever feel better after seeing their doctor. Additionally, there is some reluctance among physicians. In the public sector, doctors frequently view telemedicine as an added responsibility or workload. As a result, telemedicine must be integrated into doctors' daily tasks. Private physicians occasionally worry that telemedicine will hurt their ability to practice. They must understand that this technology expands their audience and visibility and will probably lead to further growth in their business [24].

Yet again, some medical experts are skeptical about the visual quality that is communicated during teleconsultations and tele-diagnostics. To prevent any incorrect interpretation and misdiagnosis, the image quality (color, resolution, field of vision, etc.) in teleradiology, telepathology, and teledermatology should meet international standards. Due to legal and ethical issues, many healthcare professionals continue to be reluctant to participate in telemedicine techniques. Which country's lawsuit laws - those of the patient's home country or those of the distant doctor - will be applied in the event of a cross-border teleconsultation? [27].

How remote geographical areas can be benefited?

The digital gap is a crucial factor to take into account in telemedicine because it could make inequities worse. Risk factors include a lack of access to computers or smartphones, a lack of technology literacy, and unstable internet connections. Patients with lower socioeconomic positions were less likely to finish audiovisual consultations in a recent study of patients with head and neck cancers. Due to the concentration of specialized care in urban areas, rural populations living in isolated places without internet access may not be able to benefit from virtual contact. Store-and-forward telemedicine, in which a local practitioner records diagnostics with eventual evaluation, may be considered for patients without internet access. For deaf and non-English speaking patients, effective communication is of particular significance; using skilled medical interpreters will be essential to provide fair virtual treatment [21].

The main obstacles to providing diabetic healthcare to rural areas are a lack of understanding due to illiteracy, a shortage of doctors and paramedics educated in diabetes, limited access to healthcare due to issues with transportation and infrastructure, and affordability due to poverty. Rural populations rarely check for the condition, which results in a considerably higher incidence of undiagnosed diabetes [33]. Diabetes-related problems may become more likely if treatment is inadequate or delayed. Thus, diabetes screening programs are critical in India's rural areas and other developing nations. With this objective in mind, the Chunampet Rural Diabetic Prevention Initiative was established to deliver widespread screening, diabetes healthcare, and prevention to rural India utilizing a combination of telemedicine and personalized care [1, 22]. The benefits of telemedicine for enhancing public health include lowering the cost of treatment and follow-up; improving prognosis even though access to standard treatment is accessible; maintaining databases about various diseases and locations; remotely training medical students by subject-matter experts; updating health information; reduced response times for controlling epidemics, outbreaks, or disasters; and using the telecardiology programs to screen for conditions like rheumatic heart disease [34]. 

Is telemedicine beneficial in emergency conditions?

Peritoneal dialysis patients may benefit from a well-structured telemedicine protocol if it enables evaluation of the patient's living environment, assessment of the patient's capacity to follow the recommended technique, observation and correction of potential or actual hazards that may increase the risk of infection, and reinforcement of patient confidence in self-care by providing support and encouragement. These are just a few of the potential advantages that have been highlighted. Additionally, telemedicine monitoring and home-visit programs foster a stronger bond between patient and their caretakers [35]. Diagnostics and reviews are two processes that telemedicine may help with. A mobile phone app that has been developed to make it possible for non-medical personnel to identify episodes as epileptic has good diagnostic application, sensitivity, and specificity. The usage of phone reviews or short messaging services can enhance management in a variety of ways [36].

People in low- or middle-income countries, especially those affected by war, have disproportionately high mental health burdens exacerbated by inadequate mental healthcare [37]. Afghanistan has shown the value of telehealth programs using mobile phones, such as public health initiatives to reduce stigma. Other low- or middle-income countries may follow Afghanistan's approach [38]. As mobile phones become more common, e-health or digital health, which allows people to access mental health information online, has significance for all of the world's nations. People frequently use digital platforms to find information on mental illness; thus, non-profit organizations, free helplines, and digital educational initiatives should all continue to get international support [39]. Because there may be a sense of urgency, acute sickness should ideally be evaluated by a licensed healthcare professional in person [40]. When the patient's history and physical exam can be completed "locally" and the results are electronically transmitted to a remote consultant, telemedicine has been proven to be effective in an urgent medical scenario [41].

Need for changes and further support

In March 2020, the Indian government released updated regulations for the use of telemedicine. This new public policy intends to provide additional guidance for India's telemedicine industry. More patients are using telemedicine as a result of COVID-19, and more medical settings are now using telemedicine as well. India has to address access and infrastructure problems before telemedicine is widely used. Additionally, thorough and legally binding legislation that establishes clear frameworks for doctor-patient communication to address consent, privacy, and utilization issues must be made sure to exist [42]. Even if the usage of telemedicine is increasing, to scale it up and ensure its sustainability in the long run, strong evidence regarding how telehealth might improve primary care must be developed, put into action, examined, and produced [43]. Telemedicine implementation could be strengthened by adopting integrated information systems, including stakeholders, increasing capacity, and carefully monitoring the transition. This might help telemedicine become the new benchmark for the delivery of complete medical care [44].

## Conclusions

India has been trying the use of technologies for information and communication in healthcare and education for more than 10 years. Indigenous tools and technology have improved, policy difficulties have been resolved, and national-level programs are presented through various stages of implementation. The adoption of technology into the health system will take some time since health is a state issue. The biggest challenge policymakers, governments, and implementing committees have faced during the pre-epidemic era is a lack of legislative support to promote the adoption of digital health solutions. With a new lead, broadband connection over optical fiber to remote places, the Indian prime minister's objective to transform India into a knowledge society has now been achieved. The National Medical College Network, which connects medical schools across the country for e-education, and the National Rural Telemedicine Network, which provides e-healthcare, are both part of an e-health project being carried out by the Ministry of Health and Family Welfare of the Indian government. The project has already undergone a tremendous amount of modification. The locations of the regional resource centers and the National Medical College Network have been determined. Appropriate arrangements will be made for the medical colleges that will be connected to these five centers.

Currently, a large number of national telehealth initiatives depend on terrestrial broadband connectivity rather than satellite communication. Furthermore, its greater adoption and application will help us improve pandemic readiness in the future, even in remote areas. As the population has expanded and the average lifespan has increased, a growing gap has developed between residents and those who provide basic health services. Due to a lack of primary care delivery networks and referral pathways, secondary and tertiary care facilities' health systems are overworked. The development of medical services is greatly restricted by a shortage of health workers as well as demographic and geographic issues. The elderly population change, which increases the need for remote medical care and treatment, can be solved by telemedicine. To successfully use telemedicine, proper planning and operating processes are required. Telemedicine is an innovation whose growth and widespread use have the potential to greatly affect public health. Thus, the development and implementation of telemedicine will improve patient care and India's public health system in the future. Finally, telemedicine's cost-effectiveness will likely be its most significant outcome.
